# Minimally invasive management of ureteral strictures: a 5-year retrospective study

**DOI:** 10.1007/s00345-018-2539-5

**Published:** 2018-10-30

**Authors:** C. Reus, M. Brehmer

**Affiliations:** 1 Department of Molecular Medicine and Surgery, Karolinska Institutet, Stockholm, Sweden; 2 Department of Clinical Sciences, Karolinska Institutet, Stockholm, Sweden

**Keywords:** Ureteral stricture, Minimally invasive procedure, Dilatation, Balloon dilatation

## Abstract

**Introduction:**

Ureteric strictures are well-documented complications related to surgery or radiation therapy. Minimally invasive treatment using endoscopic dilatation or laser incision is the standard practice. There are no existing guidelines on which techniques to use in the treatment of different stricture types and a paucity of data regarding long-term results.

**Purpose:**

Our study aimed to retrospectively assess the long-term efficacy of minimally invasive treatment in benign and malignant ureteric strictures.

**Materials and methods:**

Over a 5-year period, 2007–2012, we analyzed the data of 59 consecutive patients undergoing minimally invasive treatment for symptomatic ureteric strictures. We excluded 16 patients from final analysis due to failed access or loss to follow-up. All patients but one were treated with antegrade, retrograde balloon or catheter dilatations. Successful outcome was defined as an asymptomatic, completely catheter free patient, with stable renal function.

**Results:**

43 patients were eligible for retrospective final analysis. The largest proportion of strictures occurred following surgery combined with radiotherapy 8/43 (19%). Preoperative decompression was required in 30/43 (70%). We identified 32/43 (75%) balloon dilatations, 10/43 (23%) catheter dilatations and 1/43 (2%) laser incision. Overall success rate was 31/43 (72%). All 6 recurrences occurred within 36 months, 4 within the first 12 months. 3/6 patients were successfully re-dilated.

**Conclusion:**

Minimally invasive treatment is a worthwhile alternative in strictures due to previous radiation and/or surgical treatment of malignancies. Most recurrences occurred within the first year. However, late recurrences arise; therefore, patients should be subject to long-term follow-up. Moreover, re-dilatation may be required.

## Introduction

Symptoms or findings related to ureteral strictures are variable ranging from acute flank pain, sepsis with or without pyonephrosis or incidental finding of hydronephrosis. These signs may have a late presentation due to the slow development of ureteral fibrosis.

**Fig. 1:**
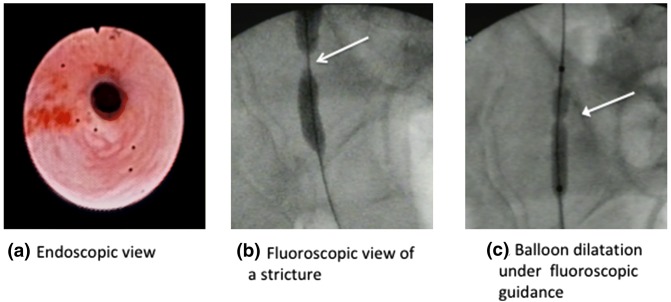
Minimally invasive management of ureteric strictures: balloon dilatation

Minimally invasive surgery is a widely used treatment for ureteral strictures. Balloon, catheter dilatation and holmium laser endoureterotomy are alternatives to open surgery (Fig. 1). However, there is little in the literature about their long-term efficacy, particularly in strictures secondary to radiation or surgery for malignancy. Most of the evidence available is based on retrospective, single-center studies with short-term follow-up [1–5].

Based on several studies on laser incision of ureteric strictures [6–17], the latest EAU Guidelines conclude that retrograde endoureterotomy should be considered as a first-line treatment option [18]. However, there are no randomized controlled studies comparing different treatment alternatives.

Factors that may have an impact on the success rate are stricture location (upper, mid or distal ureter), stricture type (ischemic versus non-ischemic, malignant or benign), stricture onset, renal function and stricture length [19]. Strictures > 2 cm have been shown to be associated with poorer outcome [2, 18].

Complex, multifactorial ureteral strictures of diverse etiologies were referred to our center, often with previously failed dilatation attempts. Our study aimed to assess the long-term efficacy of minimally invasive treatment in ureteral strictures of benign and post-malignant etiologies.

## Patients and methods

We carried out a retrospective consecutive, single-center study over a period of 5 years, from 2007 to 2012. A single surgeon performed the minimally invasive procedures in this cohort. All patients were prospectively collected using the hospital’s filing system, which combined EMR (Electronic Medical Records), ICD (International Classification of Disease) and the Swedish procedures coding system. Inclusion criteria were ureteral strictures causing pain, recurrent infections, pyonephrosis and/or renal impairment. Strictures could be of benign or post-malignant origin. Benign strictures were secondary to ureteric lithiasis, idiopathic, pelvic–ureteric junction obstruction, retroperitoneal fibrosis or urinary diversion due to neurogenic bladder disorder. Post-malignancy strictures were all due to an underlying gynecological, colorectal or urological neoplasia, previously treated by chemo/radiotherapy and/or surgery before benefitting from minimally invasive stricture management. All patients with malignancy were considered cured or in remission prior to ureteric dilatation attempt.

Preoperative work-up included radiographic investigation with CT scans with or without contrast, depending on renal function and/or MAG3-renogram (focusing on outflow obstruction and function of the affected kidney), and in one case, pre-operative magnetic resonance imaging (MRI). All patients who had a nephrostomy had an antegrade pyelography performed.

During surgery, contrast was injected with antegrade and retrograde approach, if possible, to survey the stricture. A guide wire was inserted through the stricture using retrograde or antegrade approach, under ureteroscopic guidance if needed. In most cases, the guide wire was inserted using an antegrade approach, through the ureter and out via the urethra. Pulling the wire gently rendering it taught, i.e., holding a tight grip at both antegrade and retrograde ends, facilitated balloon or dilatation catheter insertion through the stricture.

Ureteral balloon dilatation was, by default, the first intervention of choice. However, in some cases, dilatation catheters were required instead. We used a 4 cm 15F UroMax Ultra™ High Pressure Balloon Catheter **(**Boston Scientific, Natick, MA, USA) at 20 atmospheres for 3 min under perioperative fluoroscopic guidance. If a stricture was hard and tight and did not respond to dilatation with a 15-F balloon, the dilatation was completed with a 21-F balloon. As for dilatation catheters, 6–16-F Ureteral dilators (Boston Scientific, Natick, MA, USA) were used. For incision by holmium laser, we applied the effect of 1 J at 10 Hz. Immediate result was checked by performing a perioperative antegrade and/or retrograde pyelography. A 6-F ureteral double pigtail stent (Percuflex™, Boston Scientific, Natick, MA, USA) was inserted and left for 6 weeks after surgery. If the patient had a percutaneous nephrostomy tube in situ, it was clamped but left in place during these 6 weeks. An antegrade pyelography was performed 2 weeks following stent removal. If the patient did not have a nephrostomy, the first renal scintigram or CT urography was performed 2 weeks after stent removal. If the result showed improvement, however, without meeting our definition of successful treatment, a second dilatation was performed within 4 weeks.

Successful treatment was defined as a stent/nephrostomy-free patient with satisfactory passage of contrast medium on control antegrade pyelography or CT urography, or MAG-3 scintigram, and a stable renal function. Patients who did not meet these criteria after two consecutive dilatations were considered as failures.

Stent- and nephrostomy-free patients were followed up with a strict protocol including a CT urography and/or renal scintigram (MAG-3) combined with renal function evaluation with serum creatinine and estimated GFR at 3, 6, 12, 24 and 36 months.

## Results

Fifty-nine consecutive patients met the inclusion criteria. However, 16 patients were excluded, 13 of which required invasive surgery due to failed access at first dilatation attempt, i.e., the guide wire could not be passed through the stricture. The other three patients were followed up in another hospital. Consequently, 43 patients were included in the study, 22 men and 21 women. The median age at the time of surgery was 49.5 (83–16) years with a mean age of 61.1 ± 14.6 years.

As represented in Fig. 1 and Table 1, 14/43 (32%) of strictures were benign. Six cases were idiopathic and five were secondary to ureteric lithiasis. One patient developed a stricture after a pyeloplasty for a pelvic–ureteric junction obstruction and another developed a distal stricture following a urinary diversion due to a neurogenic bladder. Finally, another patient developed a stricture due to a particularly aggressive form of retroperitoneal fibrosis.

**Table 1:** Summary and results of our material. Follow-up at 6 months and at 36 months after the last dilatation

Etiology *n*/*N* (%)	Ureter location *n*/*N* (%)			Stricture length (cm)		Number of primary dilatation *n*/*N* (%)		Dilatation method *n*/*N* (%)			Success rate *n*/*N* (%)	
	P	M	D	< 2 cm	> 2 cm	1 ×	2 ×	Balloon	Catheter	Ho. laser	6 months	36 months
Benign 14/43 (32%)												
Idiopathic 6/14 (43%)	2		4	6		5	1	4	2			
Lithiasis 5/14 (36%)	2		3	5		5		3	2			
Urinary diversion for neurogenic bladder 1/14 (7%)			1	1		1		1				
Pyelo-ureteric junction obstruction 1/14 (7%)	1			1		1				1		
Collagen colitis 1/14 (7%)			1			1		1				
Total	5/14 (36%)		9/14 (64%)	14/14 (100%)		13/14 (93%)	1/14 (7%)	9/14 (64%)	4/14 (28%)	1/14(8%)	13/14 (93%)	13/14 (93%)
Post-malignant 29/43 (68%)												
Surgery and radiotherapy 8/29 (27%)		2	6	3	5	6	2	7	1			
Radical cystectomy 6/29 (21%)			6	2	4	6		6				
Radical prostatectomy 4/29 (14%)			4	2	2	3	1	2	2			
Radiotherapy 3/29 (10%)			3	2	1	2	1	1	2			
BCG/Mitomycin (UTUC) 4/29 (14%)			4	3	1	3	1	4				
Distal ureter malignancy 4/29 (14%)			4	3	1	3	1	3	1			
Total		2/29 (7%)	27/29 (93%)	15/29 (52%)	14/29 (48%)	23/29 (79%)	6/29 (21%)	23/29 (79%)	6/29 (21%)		21/29 (72%)	18/29 (62%)^a^
Overall	5/43 (12%)	2/43 (4%)	36/43 (84%)	29/43 (67%)	14/43 (33%)	36/43 (84%)	7/43 (16%)	32/43 (74%)	10/43 (23%)	1/43 (3%)	34/43 (79%)	31/43 (72%)

*D* distal ureter, *M* mid ureter, *P* proximal ureter, *Ho* holmium, *UTUC* upper tract urothelial carcinoma, *1 ×* one dilatation, *2 × *two dilatations

^a^Three recurrences failed re-dilatation

**Fig. 2:**
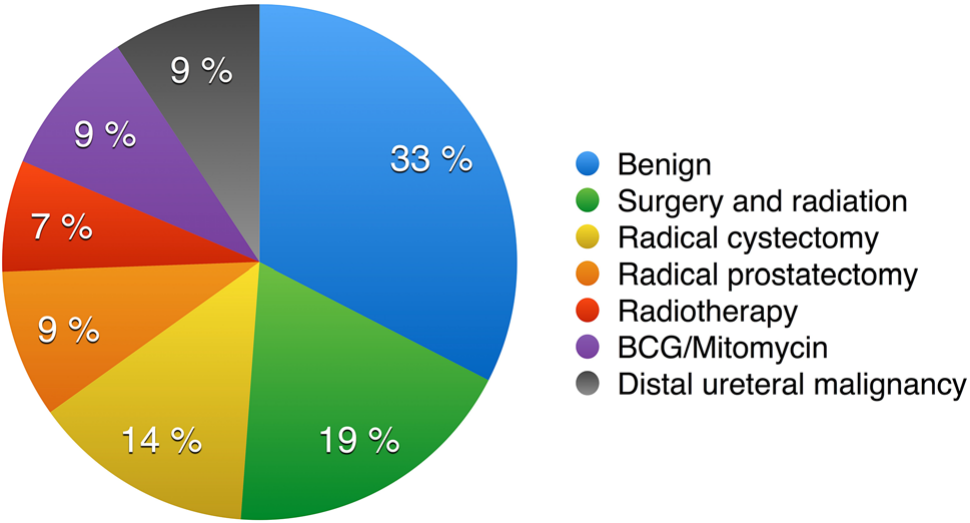
Etiologies of ureteric strictures

We identified that 29/43 (67%) of strictures were post-malignant (Fig. 2). Eight patients had developed strictures after a combination of surgery (colorectal, gynecological) and radiotherapy; six patients after radical cystectomy; four after radical prostatectomy; three after radiotherapy alone and four patients after receiving Mitomycin or BCG for ureteric transitional cell carcinoma. The remaining four patients had had concomitant bladder cancer involving the distal ureter.

Renal decompression prior to surgery was required in 30/43 (70%) of the patients. Of these, 24/30 (80%) had percutaneous nephrostomy (PCN) insertion and 6/30 (20%) had a double pigtail stent inserted.

Most patients, 32/43 (74%), were treated using retrograde balloon dilatation over a guide wire inserted using an antegrade approach. Retrograde catheter dilatation was performed in 10/43 (24%) and 1/43 (2%) underwent holmium laser incision.

Overall, 34/43 (79%) patients were initially successfully treated at 6-month follow-up. In the benign strictures group, 13/14 (93%) were successfully treated and there were no recurrences seen at 36-month follow-up. One benign case, 1/14 (7%), failed minimally invasive treatment. This was an unusual form of aggressive progressive retroperitoneal fibrosis secondary to collagen colitis. The patient required a Memokath 051™ (PNN Medical A/S, Kvistgaard, Denmark) stent insertion, for ureteral patency maintenance 12 months after the initial treatment [20–22].

Overall, within 36-month follow-up, 6/34 (17%) recurrences were observed, all of which occurred in the post-malignant strictures group. Five out of the six recurrences were initially > 2 cm long, 3/6 with a stricture length ≥ 5 cm. Of the six recurrences, four recurred within the first 12 months, one within 24 months and the other within 36 months. Three out of six recurrences (50%) were successfully re-dilated. Two re-dilated patients met successful treatment criteria at 36-month follow-up from the last dilatation, whereas one patient deceased earlier from progressive malignancy. Of the remaining three patients, one required a Memokath 051™ stent insertion [23, 24], the second a permanent nephrostomy (both due to progressive malignant disease), and the third patient was lost to follow-up. Consequently, the overall success rate at 36-month follow-up was 31/43 (72%).

Immediate failures were seen in 9/43 (21%) cases. All of these patients were considered not amenable to further surgical management. Of these, there was only one benign stricture, which was secondary to progressive collagen colitis. Of the post-malignant strictures, two were following cystectomy, one a prostatectomy, two involved surgery combined with radiotherapy, one radiotherapy, one ureteric malignancy with tumor progression and one bladder cancer with distal ureteric injury post-TURB followed by tumor progression. Of the nine failures, 2/9 (22%) had strictures of > 4 cm. In the group of failures, a permanent percutaneous nephrostomy was required in 5/9 (56%) cases. A Memokath™ stent was inserted in 3/9 (11%) patients and 1/9 (33%) benefitted from bilateral stent insertion. Our material and results are summarized in Table 1.

We had only four strictures developed following radical prostatectomy in our cohort, one open and the others by robotic-assisted technique. The open case was successfully dilated in spite of a long (5 cm) stricture. This patient was recurrence free at 6 months but needed a re-dilatation at 10 months. He was thereafter recurrence free until his death from cancer progression at 24 months after initial treatment.

Six patients developed distal ureteric strictures after radical cystectomy. Five out of six patients underwent open cystectomy and 1/6 patient robotic-assisted cystectomy with orthotropic bladder reconstruction. In the latter case the stricture was left-sided and 4 cm long. Dilatation of the stricture failed and the patient had bilateral percutaneous nephrostomy catheters inserted due to cancer progression, causing outflow obstruction.

In the open cystectomy group, two patients had strictures > 2 cm and three strictures < 2 cm. Only one failure was observed in this group; once again a left-sided 4-cm stricture combined with disease progression. This patient had an ileal conduit requiring revision of the uretero-enteric anastomosis 12 months after initial treatment. This failed and the patient kept his nephrostomy. The remaining four cases were a success at 6- and 36-month follow-up.

## Discussion

Most strictures treated in our center (29/43, 68%) were post-malignant, secondary to surgery (urology, gynecology or colorectal) or radiation, or a combination of surgery and radiotherapy, i.e., strictures requiring highly complex management. The majority required preoperative decompression. In our series, balloon dilatation was the technique of choice, used in 32/43 (74%) of our patients. Catheter dilatation was used in 10/43 (24%) patients. We used the holmium laser in one patient 1/43 (2%) with a proximal ureteral stricture secondary to an impacted lithiasis.

Complex strictures, i.e., tight, long and post-malignant strictures may need more than one treatment for a satisfactory outcome. However, more than two sessions will probably not bring any further benefit. Seven out of 34 (21%) of the successfully treated patients required two primary dilatations to achieve satisfactory ureteric contrast passage.

In spite of the high complexity of the cases, we achieved an overall success rate of 31/43 (72%). Our results were comparable to, if not better than, those described in the literature [2, 25–27]. Byun et al. report a success rate of 67 and 57% at 12 and 36 months, respectively, for benign strictures, but only 18 and 14% success rate, respectively, for a similar follow-up in malignant strictures [2]. Razdan et al. report an overall 74% success rate (for a follow-up ranging from 0.5 to 9 years) in 50 patients [26]. As for Punekar et al., an overall success rate of 69% in a 16-patient retrospective study is reported, for a follow-up period ranging from 15 to 53 months after procedure [27].

Our study underlines the latest EAU Guidelines “Grade of recommendations”, stating that patients need long-term follow-up and that late stricture recurrence may be expected up to at least 18 months postoperatively [18]. We demonstrated that stricture recurrence can even occur at 36 months and recommend a minimum of 36-month follow-up for these patients, especially in post-malignant strictures and stricture length > 2 cm. Unfortunately, ureteric strictures have a recurrent pattern. Within 36-month follow-up 6/34 (17%) patients had recurred. However, 3/6 (50%) of recurrent strictures were successfully re-dilated; the remaining half presented with progressive malignant disease, no longer amenable to minimally invasive approach.

Immediate failures were observed in 9/43 (21%) of the patients. All but one were complex malignant cases with progressive disease. The only benign case was a patient with rapid progression of a retroperitoneal fibrosis secondary to collagen colitis. This study also showed that strictures of > 2 cm may be successfully treated with dilatation. We had two patients who fully responded to treatment, in spite of their ureteric stricture length of > 4 cm. Despite the complexity and the advanced nature of the strictures, the overall success rate was high. Our results highlight that minimally invasive treatment is always worth attempting, even if the stricture may appear radiologically challenging. Our study demonstrated that balloon dilatation may be recommended as treatment of choice.

Although our center is a high-volume reference center for cystectomy and prostatectomy, we had very few strictures from this particular patient group, which is comparable to the previously published studies [28]. All strictures in the cystectomy and prostatectomy groups were distal, most probably due to ischemia, following extensive dissection.

Moreover, more strictures were observed after open radical cystectomy, in line with current publications [29]. These strictures, as those observed post-prostatectomy, were presumably secondary to ischemia in the context of extensive dissection. It is noteworthy that these cases responded well to minimally invasive management.

The limits of this study are its retrospective, single-center nature and its small number of patients. A randomized controlled, multi-centric study needs to be carried out.

## Conclusion

Minimal invasive treatment, preferably using balloon dilatation in the first instance or catheter dilatation, is an alternative well-worth attempt in benign and post-malignancy strictures if access is feasible. In this study, the overall success rate was 72% at 36-month follow-up. Dilatation may need to be repeated to achieve success, at initial treatment as well as in late recurrences. Strictures may recur after more than 30 months. Patients benefit from long-term follow-up in adherence to a strict protocol.
